# Cementoblastoma With Bony and Soft Tissue Recurrences Following a Primary Molar Lesion: A Case Report

**DOI:** 10.1155/crid/8903042

**Published:** 2026-10-27

**Authors:** Eman Allam, Craig C. Thiede, Yi-Ling Lin

**Affiliations:** ^1^ Section of Oral Medicine Oral Pathology and Orofacial Pain UCLA School of Dentistry Los Angeles California USA ucla.edu; ^2^ Section of Public and Population Health UCLA School of Dentistry Los Angeles California USA ucla.edu; ^3^ Private Practice and Children′s Hospital of Orange County Orange California USA; ^4^ Gene Regulation Program UCLA Jonsson Comprehensive Cancer Center Los Angeles California USA ucla.edu; ^5^ Department of Pathology and Laboratory Medicine David Geffen School of Medicine at UCLA Los Angeles California USA ucla.edu

**Keywords:** cementoblastoma, odontogenic tumor, recurrence

## Abstract

Cementoblastoma is a rare benign odontogenic neoplasm defined by its fusion to the root of a tooth and characterized by radiating cementoid deposition with peripheral active cementoblasts. Although recurrence is uncommon following complete excision, when it occurs, it typically arises at the original site. We report an unusual recurrent cementoblastoma associated with a primary mandibular molar in a pediatric patient. Review of records from the initial presentation documented a radiopaque mandibular lesion fused to the roots of the lower right primary first molar with cortical expansion, which was treated by extraction of the involved tooth and removal of the associated lesion. Eighteen months later, the patient presented with recurrence as multiple discrete, noncoalescent masses involving the alveolar crest, lingual cortical bone, and buccal gingival soft tissue. All recurrent lesions, including the soft tissue lesion, showed histologic features consistent with developing cementoblastoma. Each lesion demonstrated a radial growth pattern forming a well‐circumscribed spherical mass. This case documents recurrence after apparently conservative initial removal of a cementoblastoma arising in the primary dentition, and supports the concept that neither persistent root attachment nor an osseous environment is required for lesion development or for maintaining the characteristic radial growth pattern. The findings highlight the importance of complete excision at initial presentation to reduce the risk of recurrence.

## 1. Introduction

Cementoblastoma is a rare odontogenic neoplasm that most commonly arises in the posterior mandible, often in association with the permanent first molar. It typically presents in patients during the second/third decade of life and shows no significant gender predilection. Unerupted and deciduous teeth are rarely affected. The outline of the roots of the involved tooth is usually obscured by root resorption and fusion of the tumor with the tooth [1–5].

Radiographically, cementoblastoma typically presents as a well‐defined radiopaque mass fused to the tooth root and surrounded by a narrow radiolucent rim continuous with the periodontal ligament. Histologically, it is characterized by cellular cementum with prominent reversal lines, active cementoblasts, and peripheral radiating columns of cementoid within a fibrovascular stroma [1, 4, 6]. These clinicoradiographic and histologic features help distinguish cementoblastoma from other periapical radiopaque lesions, including cemento‐osseous dysplasia, condensing osteitis, and hypercementosis [5].

Although historically considered a lesion with a low recurrence rate, subsequent reviews suggest that recurrence may be more frequent than previously recognized [3, 7]. We report an unusual case of recurrent cementoblastoma arising from a primary tooth, presenting as multiple calcified nodules involving separate intraosseous and soft‐tissue sites, including the alveolar crest, lingual cortical bone, and adjacent buccal gingiva. Cementoblastoma involving primary dentition is uncommon, and soft‐tissue recurrence is particularly rare. The characteristic radial growth pattern was preserved across recurrent sites, despite differences in root attachment and osseous environment.

## 2. Case Presentation

A 7‐year‐old girl presented with recurrent swelling of the right mandible. Review of original records revealed that 18 months earlier, she had presented with a 2‐week history of right mandibular swelling and underwent surgical removal of a lesion involving the lower right primary first molar. The original operative report described a firm, round solid tumor associated with the lower right primary first molar, with expansion of both the buccal and lingual cortical plates. During the initial procedure, the involved tooth was extracted together with the associated tumor. Original imaging from the first presentation demonstrated a calcified/radiopaque lesion centered on and fused to the roots of the involved primary molar. The lesion was reportedly diagnosed elsewhere as an osteoma with associated osteomyelitis (Figure 1).

**Figure 1:**
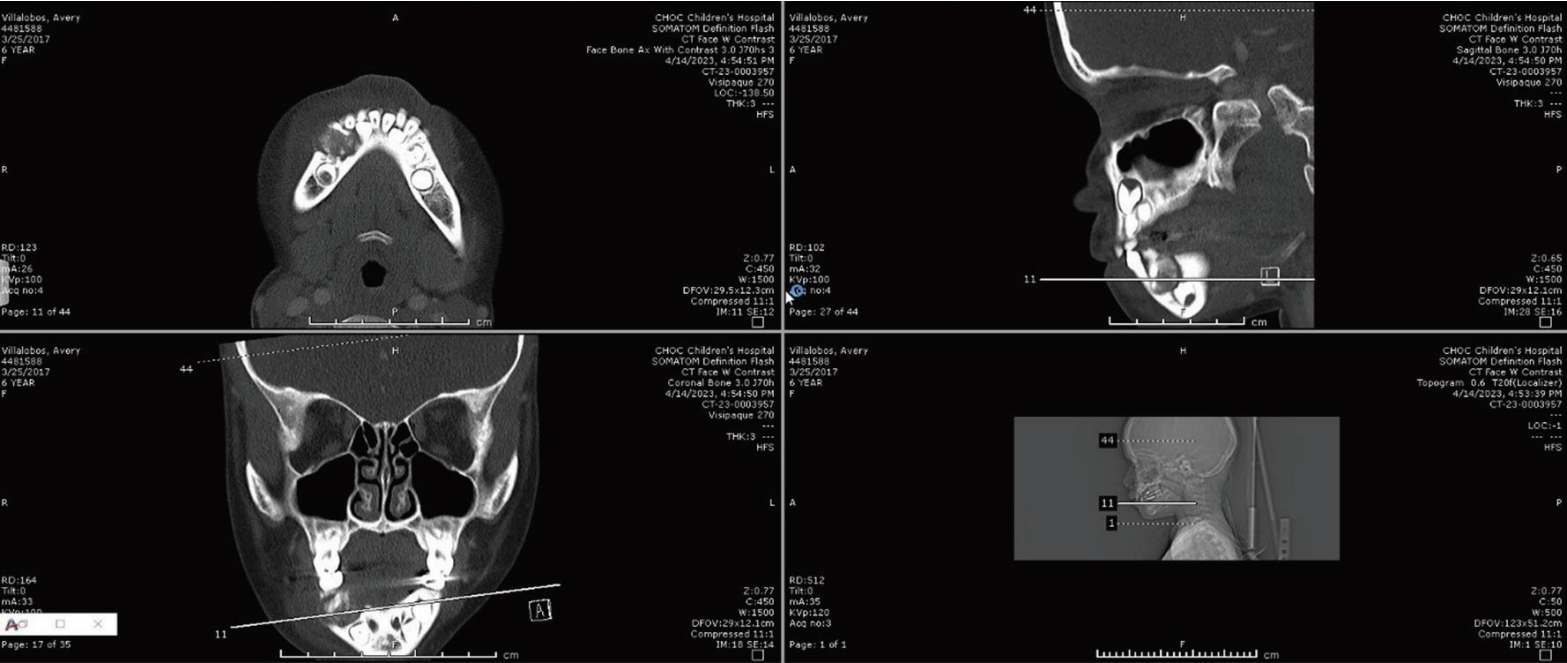
CBCT images from the initial presentation at the age of 6 years demonstrated a well‐defined radiopaque lesion associated with and fused to the roots of the lower right primary first molar, with expansion of the adjacent cortical plates.

At the current evaluation, CBCT demonstrated multiple discrete calcified masses, including two medium‐sized lesions along the lingual cortical bone and one large lesion within the buccal gingiva (Figure 2). The initial clinical impression was a fibro‐osseous lesion. The patient subsequently underwent surgical excision of the recurrent lesions, during which an additional small lesion was identified at the alveolar crest. All four lesions were removed and submitted for histopathologic examination. Grossly, the specimen consisted of multiple calcified masses measuring 15 × 10 × 6 mm in aggregate.

**Figure 2:**
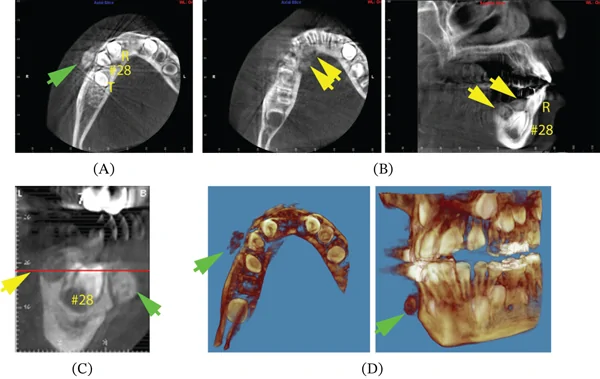
CBCT demonstrating multiple round, well‐circumscribed radiopaque masses. (A) Axial image demonstrates a calcified mass (green arrowhead) buccal to the primary canine (R), the unerupted developing first premolar (#28), and the primary second molar (T). (B) Axial (left) and sagittal (right) images demonstrate two calcified masses (yellow arrowhead) within the lingual alveolar ridge, each with a surrounding radiolucent rim. (C) Coronal image demonstrates one calcified mass buccal (green arrowhead) and another lingual (yellow arrowhead) to the developing first premolar (#28); the buccal mass corresponds to that shown in (A), and the lingual mass corresponds to one of the lesions shown in (B). (D) Three‐dimensional reconstructions of the buccal mass are shown. Left: axial view of the isolated mandible, corresponding to (A). Right: frontal view with slight leftward rotation to avoid mandibular superimposition.

Histologically, all masses were well‐circumscribed and spherical, with an eosinophilic peripheral border rimmed by plump cementoblasts arranged in a characteristic radiating pattern (Figures 3 and 4). Centrally, the lesions consisted of a prominent fibrovascular stroma containing trabeculae of cellular cementum and numerous microfoci of cementum matrix. The cementum trabeculae exhibited enlarged lacunae containing cementocytes, lined by plump cementoblasts, and showed readily apparent osteoclastic activity. The lesion displayed marked variability in mineralization, with ill‐defined basophilic zones and focal reversal lines. A peripheral‐to‐central mineralization gradient was evident, characterized by predominantly poorly mineralized cementum matrix at the periphery and increasing, yet variable, mineralization centrally. The microfoci of cementum matrix were poorly mineralized, whereas the more mineralized trabeculae remained variably organized, with some demonstrating distinct reversal lines and others exhibiting ill‐defined basophilic zones.

**Figure 3:**
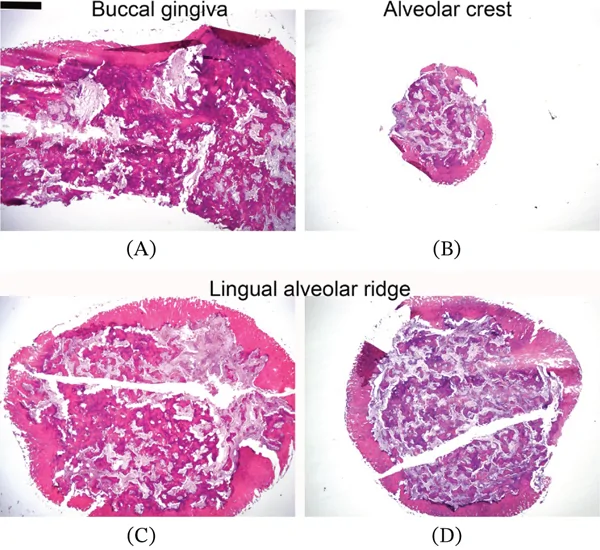
H&E‐stained low‐power micrographs of recurred cementoblastomas from four locations, demonstrating well‐circumscribed, spherical masses of variable sizes. The low‐power views highlight the overall architecture of the lesions. (A) Largest buccal soft‐tissue lesion. (B) Smallest alveolar crest lesion (not visible on CBCT). (C,D) Two medium‐sized lingual lesions. Original magnification ×20; scale bar = 1 mm.

**Figure 4:**
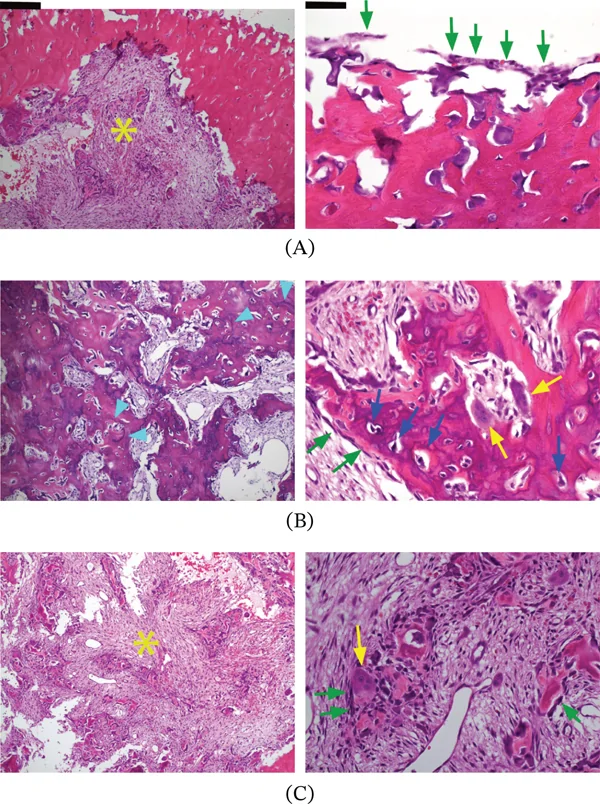
H&E‐stained high‐power micrographs of recurrent cementoblastomas. Left: ×100, scale bar = 200 *μ*m; right: ×400, scale bar = 50 *μ*m. (A) Abundant fibrovascular tissue (yellow asterisks) along with peripheral radiating columns with plump cementoblastic rimming (green arrows). (B) Trabeculae of cementum with variable degrees of mineralization and reversal lines (cyanide arrowheads), containing enlarged lacunae with cementocytes (blue arrows) as well as conspicuous cementoblasts (green arrows) and osteoclasts (yellow arrows). (C) Abundant fibrovascular stroma (yellow asterisks) with microfoci of cementum matrix, associated with active cementoblastic (green arrows) and osteoclastic activity (yellow arrows).

Correlation of the recurrent lesion with the original records from the initial surgery, including the documented fusion of the original mass to the roots of the primary first molar, extraction of the involved tooth with the attached solid tumor, and the current characteristic histomorphology, supported a diagnosis of recurrent cementoblastoma. The patient underwent regular clinical follow‐up from the time of surgery through 1.5 years postoperatively, including early postoperative assessments and subsequent evaluations at approximately 6‐month and annual intervals. No further recurrence has been reported in the 1.5 years following the second surgery.

## 3. Discussion

Cementoblastoma is a benign odontogenic neoplasm that is generally well‐circumscribed and associated with favorable outcomes. Although most lesions behave in an indolent manner, inferior displacement of the mandibular canal, progressive jaw expansion, root resorption, and occasional cortical perforation have been reported [3]. Complete surgical removal with extraction of the involved tooth remains the standard management, as the tumor is characteristically fused to the root surface. When entirely removed, the prognosis is excellent [3, 7]. To our knowledge, this represents one of very few reported cases of multifocal recurrence involving both intraosseous and soft tissue locations following cementoblastoma associated with a primary tooth.

The clinical, radiographic, and operative records from the patient′s initial presentation provide important context for interpreting the recurrence. The original lesion was associated with the lower right primary first molar and was removed together with the tooth. Operative documentation described a firm, round solid tumor with buccal and lingual cortical expansion, and the original imaging supported fusion of the lesion to the roots of the involved primary molar. However, the available records did not provide sufficient technical detail to establish whether the original lesion was completely excised or whether microscopic residual disease remained. Detailed documentation regarding the extent of curettage or peripheral bone removal and the histopathologic evaluation of the initial surgical specimen was not available. Therefore, the mechanism underlying the subsequent multifocal recurrence cannot be determined with certainty.

The diagnosis of recurrent cementoblastoma was established through clinicopathologic correlation, primarily based on the characteristic histomorphologic features of the current specimens. The principal histopathologic mimics include osteoblastoma and osteoid osteoma, which demonstrate overlapping histomorphologic characteristics and shared pathogenetic mechanisms, including FOS gene rearrangements and c‐FOS overexpression [8–10]. The present lesions demonstrated a well‐organized, circumferential peripheral growth front composed of radiating columns of cementoid rimmed by active cementoblasts. This architectural pattern represents a defining feature of cementoblastoma and is not a recognized hallmark of osteoblastoma or osteoid osteoma [1, 6]. Although an earlier report [11] suggested that osteoblastoma may exhibit peripheral radiating spicules, the extent and organization of this feature were not clearly defined, nor was it demonstrated as a continuous circumferential pattern surrounding the tumor mass. In addition, the size of the current lesions is unusual for osteoblastoma, which is typically larger than 2 cm in diameter [1]. Moreover, this feature has not been consistently documented in larger osteoblastoma or osteoid osteoma series [12–15]. Instead, these studies describe osteoblastoma as exhibiting a more irregular or poorly defined peripheral architecture lacking a structured growth front, whereas osteoid osteoma is characterized by a central nidus associated with surrounding reactive host sclerosis. Although these features may be less pronounced or absent in gnathic lesions, the presence of a characteristic, well‐organized radiating cementoid peripheral zone in the current specimens strongly supported the diagnosis of cementoblastoma.

Other entities considered in the radiographic differential diagnosis included benign fibro‐osseous lesions (BFOLs), odontoma, and central osteoma. Histologically, an early‐stage BFOL could potentially mimic recurrent cementoblastoma following tooth extraction. However, with the exception of cemento‐osseous dysplasia, multifocal involvement would be highly unusual among the other BFOLs. Although immunohistochemistry (IHC) is not routinely required for the diagnosis of cementoblastoma, c‐FOS IHC may be useful in challenging cases to help distinguish cementoblastoma from BFOLs. In addition, BFOLs typically exhibit a cellular fibrous stroma, a feature that was not present in the current case. Odontoma and central osteoma are likewise histologically incompatible with the findings. An odontoma is composed of at least two dental hard tissues, whereas an osteoma consists of mature, relatively inactive compact or cancellous bone. None of the aforementioned entities demonstrate the characteristic peripheral radiating cementoid formation observed in the present case. The initial diagnosis of “osteoma with associated osteomyelitis” likely reflects the histologic overlap between cementum and bone. Cellular cementum may resemble viable bone because of its lacunar architecture, whereas acellular cementum lacks lacunae and may be misinterpreted as necrotic bone. Taken together, the clinical, radiographic, and histopathologic findings favored recurrent cementoblastoma over these differential diagnoses.

In contrast to the classic presentation of mature cementoblastoma, characterized by central consolidation into densely mineralized sheets with prominent reversal lines and limited fibrovascular tissue confined to narrow intertrabecular clefts, the current lesions exhibited a relatively prominent fibrovascular stroma. The cellular cementum was arranged as interconnecting trabeculae with enlarged lacunae housing cementocytes, lined by plump cementoblasts and accompanied by numerous osteoclasts. Although reversal lines were evident, many areas instead displayed ill‐defined basophilic zones within the cementum trabeculae, indicating a less organized pattern of mineralization. In addition, numerous microfoci of cementum matrix associated with active cementoblastic and cementoclastic activity were evident. Collectively, these features supported interpretation of the lesions as immature (developing) cementoblastoma.

Soft tissue recurrence of cementoblastoma is distinctly uncommon. In the present case, the recurrent lesions, including the soft tissue lesion, retained the characteristic radial growth pattern with a peripheral radiating border of cementoid, despite the absence of direct root fusion. Preservation of this architectural organization suggests that the radial growth pattern represents an intrinsic property of the neoplastic cementoblasts rather than a phenomenon strictly dependent upon root surface attachment or bone environment. These findings further support the view that cementoblastoma is a true odontogenic neoplasm with autonomous proliferative potential even in soft tissue.

Recurrent cementoblastoma is typically confined to the original site, whereas dissemination into separate intraosseous and soft tissue locations is highly unusual [16, 17]. In addition to the present case, Oya et al. [18] previously described a recurrent cementoblastoma exhibiting a multifocal/multicentric growth pattern with multiple radiographically distinct masses. Interestingly, their recurrent lesion demonstrated unusual histopathologic features, including cytologic atypia and a well‐demarcated sclerotic border rather than the characteristic circumferential radiating peripheral matrix emphasized in the current WHO description of cementoblastoma.

In the present case, one possible explanation of the multifocal distribution is intraoperative displacement of microscopic tumor fragments; however, residual microscopic disease or multifocal local recurrence cannot be excluded. This pattern highlights the importance of complete surgical removal, including extraction of the associated tooth and careful curettage or peripheral ostectomy when clinically indicated. Close postoperative surveillance with periodic radiographic follow‐up is particularly advisable in pediatric patients because active bone remodeling may obscure subtle residual disease and facilitate recurrence.

## 4. Conclusion

This case highlights the necessity of meticulous surgical management to prevent recurrence and documents a rare multilocular and extraosseous recurrence of cementoblastoma in a pediatric patient. The preserved radial architectural pattern provides further evidence that cementoblastoma represents a true odontogenic neoplasm with possible intrinsic morphogenetic tendencies and is potentially capable of growth independent of direct root attachment or specific osseous environment. Comprehensive clinicopathologic and radiographic evaluation is critical for distinguishing cementoblastoma from other benign osseous lesions with overlapping features. In addition to the lesion′s intimate fusion to the tooth root, the current WHO description emphasizes characteristic morphologic findings, including a circumferential radiating peripheral matrix rimmed by plump cementoblasts and the absence of a significant fibro‐osseous component. Careful recognition of these combined clinicopathologic features is important in establishing the diagnosis and differentiating cementoblastoma from nonodontogenic bone lesions such as osteoblastoma. Misdiagnosis may result in inadequate surgical management, incomplete lesion removal, and an increased risk of recurrence.

## Author Contributions

Eman Allam: data curation, writing—original draft. Craig C. Thiede: investigation and methodology. Yi‐Ling Lin: conceptualization, validation and writing—review and editing.

## Funding

No funding was received for this manuscript.

## Ethics Statement

This study was conducted in accordance with the Declaration of Helsinki and was approved by the UCLA Institutional Review Board (IRB #17–000721). Written informed consent for publication was obtained from the patient′s parent/legal guardian.

## Conflicts of Interest

The authors declare no conflicts of interest.

## Supporting information


**Supporting Information** Additional supporting information can be found online in the Supporting Information section. File S1: CARE checklist.

## Data Availability

The data that support the findings of this study are available on request from the corresponding author. The data are not publicly available due to privacy or ethical restrictions.
